# CellPalmSeq: A curated RNAseq database of palmitoylating and de-palmitoylating enzyme expression in human cell types and laboratory cell lines

**DOI:** 10.3389/fphys.2023.1110550

**Published:** 2023-01-24

**Authors:** Angela R. Wild, Peter W. Hogg, Stephane Flibotte, Shruti Kochhar, Rocio B. Hollman, Kurt Haas, Shernaz X. Bamji

**Affiliations:** ^1^ Bamji Lab, Department of Cellular and Physiological Sciences, Life Sciences Institute and Djavad Mowafaghian Centre for Brain Health, Vancouver, BC, Canada; ^2^ Life Sciences Institute Bioinformatics Facility, University of British Columbia, Vancouver, BC, Canada

**Keywords:** palmitoylation, ZDHHC, depalmitoylating enzyme, cancer, cell line, human, RNAseq, expression

## Abstract

The reversible lipid modification protein S-palmitoylation can dynamically modify the localization, diffusion, function, conformation and physical interactions of substrate proteins. Dysregulated S-palmitoylation is associated with a multitude of human diseases including brain and metabolic disorders, viral infection and cancer. However, the diverse expression patterns of the genes that regulate palmitoylation in the broad range of human cell types are currently unexplored, and their expression in commonly used cell lines that are the workhorse of basic and preclinical research are often overlooked when studying palmitoylation dependent processes. We therefore created CellPalmSeq (https://cellpalmseq.med.ubc.ca), a curated RNAseq database and interactive webtool for visualization of the expression patterns of the genes that regulate palmitoylation across human single cell types, bulk tissue, cancer cell lines and commonly used laboratory non-human cell lines. This resource will allow exploration of these expression patterns, revealing important insights into cellular physiology and disease, and will aid with cell line selection and the interpretation of results when studying important cellular processes that depend on protein S-palmitoylation.

## 1 Introduction

Protein *S*-palmitoylation is a reversible post-translational modification, in which the 16-carbon fatty acid, palmitate, is attached to a thiol group of specific cysteine residues *via* a labile thioester bond. As the most common form of *S*-acylation, this modification has profound influence over protein function and cellular signaling in the brain (Globa and Bamji, 2017; Matt et al., 2019; Ji and Skup, 2021), immune system (Lin, 2021; Zhang et al., 2021) and cardiovascular system (Essandoh et al., 2020). Furthermore, dysregulated *S*-palmitoylation is also associated with metabolic disorders (Qu et al., 2021), brain disorders (Zaręba-Kozioł et al., 2018) and viral infection (e.g., SARS-CoV-2) (Abdulrahman et al., 2021; Wu et al., 2021; Li et al., 2022). In recent years, considerable evidence has accumulated that *S*-palmitoylation of oncogenes can play a critical role in cancer progression, with the palmitoylating and depalmitoylating enzymes being investigated as drug targets to modify tumor growth (Ko and Dixon, 2018). *S*-palmitoylation is mediated by a family of 24 human ZDHHC enzymes that differ structurally but share a consensus ‘DHHC’ catalytic motif (Malgapo and Linder, 2021; Puthenveetil et al., 2022). These enzymes can associate with several known ZDHHC accessory proteins that regulate their function and stability (Salaun et al., 2020). Furthermore, depalmitoylation is mediated by a growing number of acyl-protein thioesterase enzymes, which are still in the process of being fully characterized (Won, Cheung See Kit and Martin, 2018). However, the precise distribution of these enzymes within the broad range of cell types and tissues in the human body has not been characterized, and is often overlooked when using model systems to study biological and pathological processes that are regulated by *S*-palmitoylation.

Cell lines are the workhorse of many laboratories and are often used for basic and clinical research in place of primary cell culture. In addition to being cost effective, easy to use, and generating relatively reproducible data, cells lines also bypass the need for ethical approval that is associated with human and non-human animal tissue. Furthermore, human cancer cell lines that share many transcriptomic similarities with primary tumors (Barretina et al., 2012), are the primary preclinical model systems for the investigation of cancer biology and testing the efficacy of anticancer drugs (Gonzalez-Nicolini and Fussenegger, 2005).

In addition to the numerous cancer cell lines that are central to cancer research, alternative animal cell lines are used across a huge variety of research disciplines, in fields as diverse as primary research, vaccine production, study of gene function, protein production and drug toxicity research (Allen et al., 2008; Khan, 2013; Genzel, 2015) (Allen et al., 2008; Khan, 2013; Genzel, 2015). However, the influence of the background transcriptome of a given cell line is not always taken into account when utilizing these models to study cellular processes.

The recent publication of numerous large scale RNAseq studies has enabled the transcriptional profiling of a large variety of tissues, including from human samples, animal model systems and immortalized cell lines. Importantly, this wealth of expression data is publicly available through web resources such as the Gene Expression Omnibus (GEO) and other purpose designed web resources. However, there are often barriers to easy access of this data, particularly when web resources for data viewing are not available.

A recent study from our lab collated and analyzed publicly available RNAseq data revealing considerable heterogeneity in the expression patterns of the genes that regulate *S*-palmitoylation in the mouse brain, and demonstrated how these expression patterns can be used to better understand the etiology of diseases that are related to dysregulated *S*-palmitoylation, and to predict and validate enzyme-substrate interactions (Wild et al., 2022). However, a resource for non-brain RNAseq data in human tissues and commonly used cell lines is currently lacking. We have therefore applied a similar approach to RNAseq data curation and visualization for genes that regulate *S*-palmitoylation, this time assessing their expression patterns in human bulk and single-cell RNAseq datasets, in addition to human and non-human cell lines that are a mainstay of basic biological and preclinical research. To create an accessible resource for visualizing the expression of the *S*-palmitoylationg and depalmitoylating enzymes across a vast number of cell lines, we curated data from several large scale RNAseq databases that cover hundreds of human cancer cell lines, and also curated GEO expression data from several of the most commonly used non-human and human laboratory cell lines. We then created a webtool (CellPalmSeq; https://cellpalmseq.med.ubc.ca) to allow easy interrogation of this multi-study data using simple interactive heatmaps.

## 2 Results

### 2.1 Creation of CellPalmSeq, an interactive database and webtool for visualization of the expression patterns of the genes that regulate *S*-palmitoylation

In recent years, numerous RNAseq studies of varying scales have characterized the transcriptomes of human tissues and hundreds of commonly used cell lines. However, this data is often difficult to access and visualize, with no single resource existing with transcriptomic data from a large number of non-human cell lines. Furthermore, datasets from individual studies are challenging for non-bioinformaticians to access, and available webtools often do not allow comparison of the expression of multiple genes at once. We therefore created “CellPalmSeq”, an interactive resource for visualization of expression data for the genes that regulate *S*-palmitoylation, curated from multi-study RNAseq datasets from non-diseased human tissues, human cancer cell lines and commonly used non-human laboratory cell lines. We processed data for the 24 ZDHHC enzymes (*ZDHHC1*-*ZDHHC24*, plus *ZDHHC11B* and omitting *ZDHHC10* which is not included in the ZDHHC genes), the known ZDHHC accessory proteins (*GOLGA7*, *GOLGA7B* and *SELENEOK*; Salaun et al., 2020) and the depalmitoylating enzymes that have been well characterized and/or are inhibited by the depalmitoylating enzyme blockers (HDFP) and Palmostatin B (*LYPLA1, LYPLA2, PPT1, ABHD4, ABHD6, ABHD10, ABHD12, ABHD13, ABHD16A, ABHD17A, ABHD17B, ABHD17C;* Lin et al., 2017). To compare across datasets, we present all datasets in the same expression units and reanalyzed from the raw reads where needed. All data are freely available for download on CellPalmSeq, so that users can replot data in their preferred format. We then utilized this resource to study the expression patterns of this gene family in order to highlight the importance of accounting for their expression when studying cellular signaling in different cell types, tissues, and cellular model systems.

### 2.2 Expression of the genes that regulate *S*-palmitoylation in human single cell types

**FIGURE 1:**
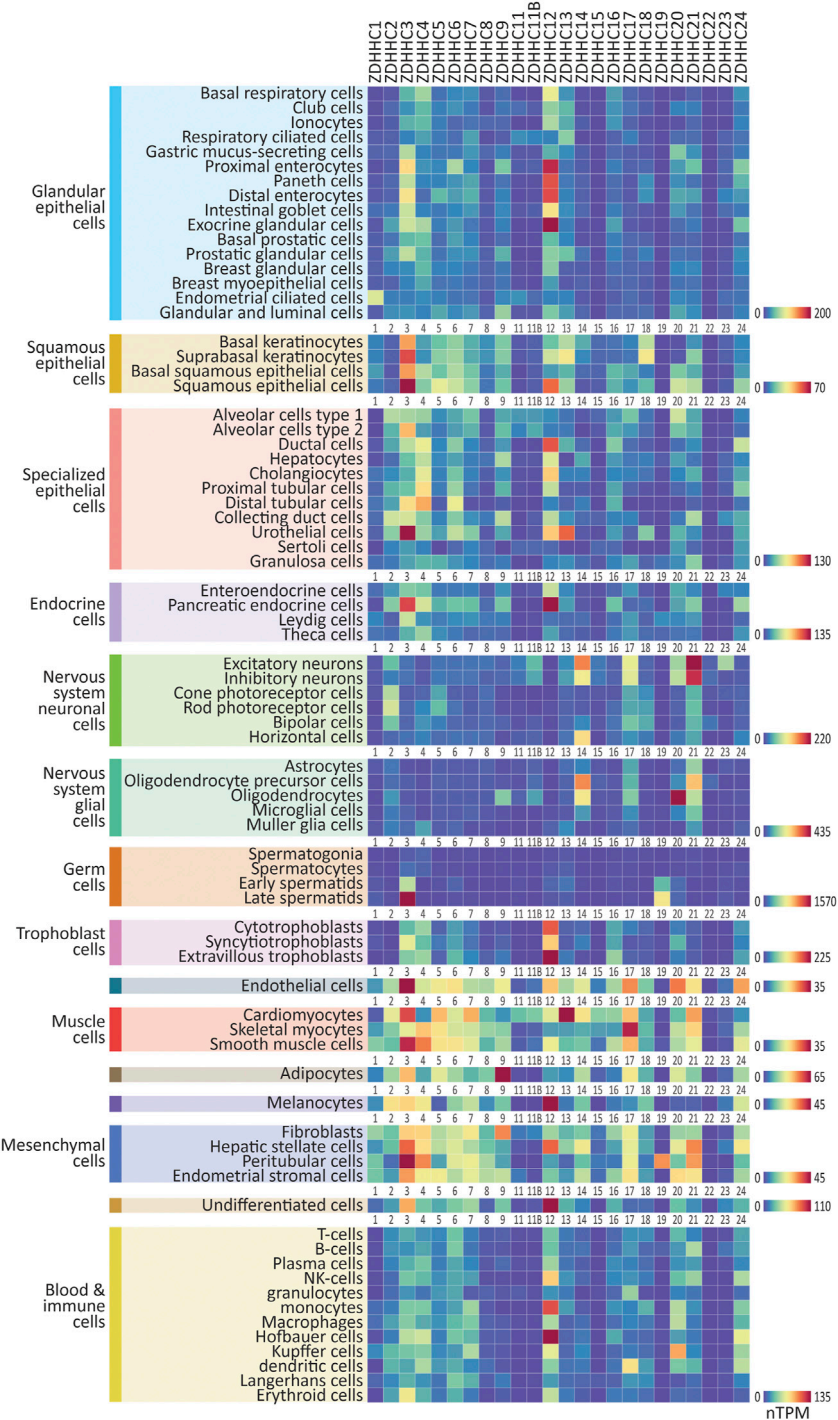
Expression patterns of the ZDHHC enzymes in major human cell types. Heatmaps showing human single-cell RNAseq expression data for the ZDHHC enzymes, downloaded from the Human Protein Atlas. 76 major cell types were identified within 15 cell type clusters. Heatmaps are grouped according to cell type cluster to view relative expression. Heatmap units: TPM. All data available for download on CellPalmSeq.

**FIGURE 2:**
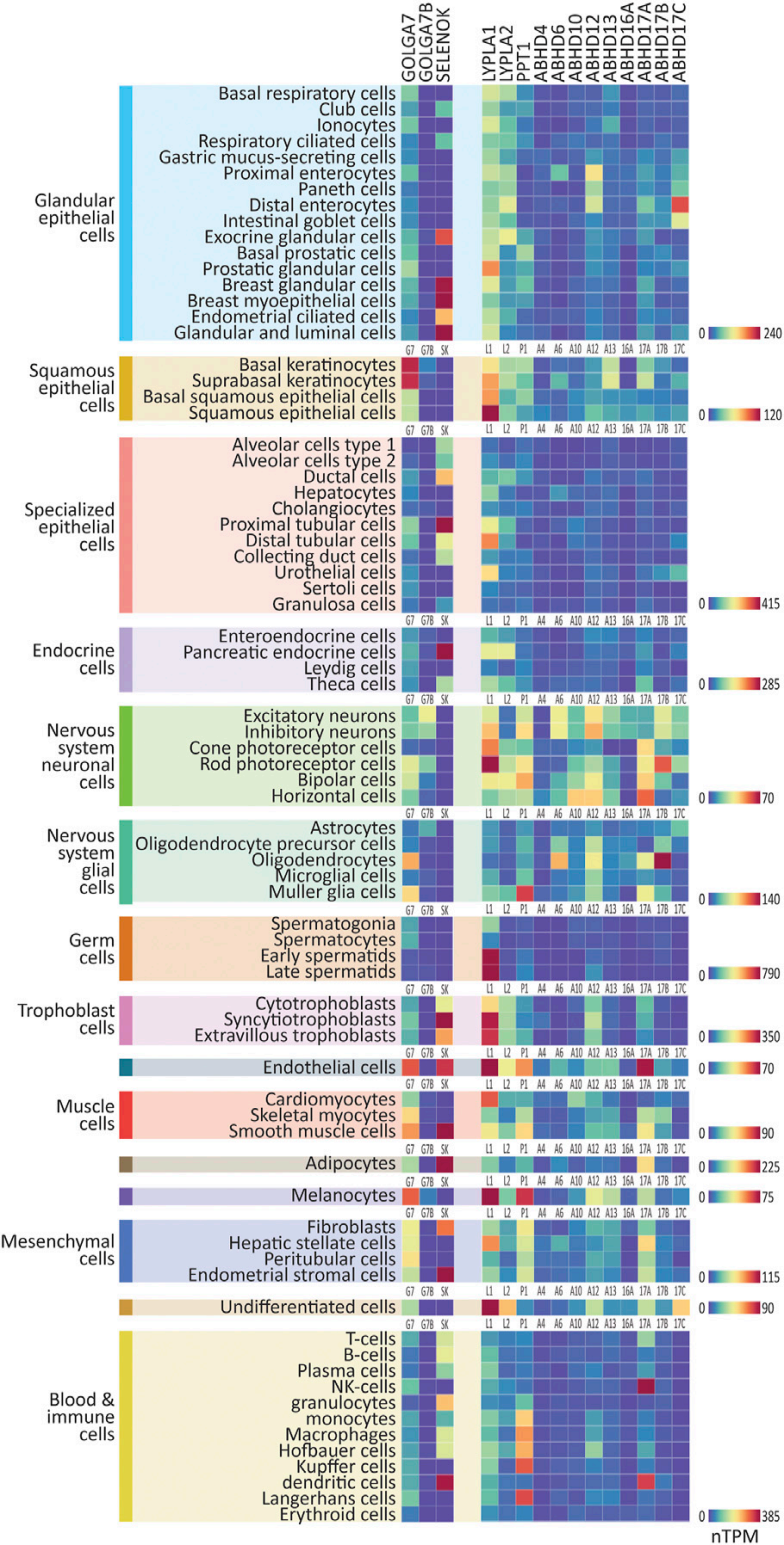
Expression patterns of the ZDHHC accessory proteins and depalmitoylating enzymes in major human cell types. Heatmaps showing human single-cell RNAseq expression data for the ZDHHC accessory proteins and depalmitoylating enzymes, downloaded from the Human Protein Atlas. 76 major cell types were identified within 15 cell type clusters. Heatmaps are grouped according to cell type cluster to view relative expression. Heatmap units: Transcripts per million (TPM). All data available for download on CellPalmSeq.

We first curated single-cell RNAseq data from the Human Protein Atlas, which analyzed collated data from 26 studies of cells from non-diseased human tissues and organs, identifying 76 broad single-cell types, divided into 15 cell type clusters (https://www.proteinatlas.org/; Karlsson et al., 2021). Single-cell RNAseq data reveal the gene expression profile of a particular cell type, as opposed to bulk tissue which is composed of multiple cell types. We downloaded expression data (normalized transcripts per million; nTPM) for the genes that regulate *S*-palmitoylation from this dataset and plotted heatmaps of ZDHHC expression. We found initially that a small number of the ZDHHC genes were expressed considerably higher than others, making visual comparison between the majority of genes difficult within a single heatmap (notably *ZDHHC3* and *ZDHHC19* in late spermatids, see CellPalmSeq for interactive heatmaps). Therefore, we plotted individual heatmaps of ZDHHC expression *within each cell-type cluster*, with heatmaps scaled separately for each cell type cluster (Figure 1). Overall, when looking at expression by cell type cluster, we observed highly heterogeneous expression, in line with specific functions of the various components of the *S*-palmitoylation machinery in different cellular contexts. Certain cell type clusters showed a preference for a very select group of ZDHHC enzymes, whereas others expressed a broader range of ZDHHC enzymes. On average, *ZDHHC3* was the highest expressing ZDHHC enzyme across all cell types, followed by *ZDHHC12*, *ZDHHC21,* and *ZDHHC4*. For the ZDHHC accessory proteins (Figure 2), *GOLGA7* was broadly expressed within the majority of cell type clusters, whereas *SELENOK* expression was highly heterogeneous. For the depalmitoylating enzymes (Figure 2), *LYPLA1* had the highest expression on average and was expressed highly in a number of cell types such as squamous epithelial cells, cardiomyocytes, and hepatic stellate cells, while *PPT1* was the depalmitoylating enzyme with the highest expression in immune cells. Interactive heatmaps for this (and all other) data are available on CellPalmSeq to plot expression data according to user preferences.

Next, to visualize the relative expression of each individual ZDHHC enzyme *across all cell types*, we plotted z-scores for each ZDHHC enzyme, calculated from expression values across all cell types examined (Supplementary Figure S1). A positive or negative z-score indicates enriched or depleted expression, respectively, in a given cell type. Several ZDHHCs were highly enriched in certain cell types, for example, ZDHHC19 in late spermatids and ZDHHC16 in extravillous trophoblasts. Depalmitoylating enzymes showed similarly enriched expression, including *ABHD17A* in NK cells, and *ABHD17B* in oligodendrocytes (Supplementary Figure S2). For the accessory proteins, *GOLGA7B* was highly enriched in cells of the nervous system, particularly neuronal cells, mirroring previous findings of enrichment of *Golga7b* in mouse neuronal cells (Supplementary Figure S2; Wild et al., 2022).

In addition to the single-cell RNAseq data presented here, bulk RNAseq data of the major human tissues curated from the Human Protein Atlas are also available on the CellPalmSeq website.

Together, these expression patterns give insight into the specialized roles that certain palmitoylating and depalmitoylating enzymes are likely to have in different cell types within the human body, and can be used to make predictions about potential substrate interactions between this machinery and cell type enriched substrates (Wild et al., 2022). Furthermore, these patterns of enrichment will aid in the understanding of potential off target effects of drugs developed to modify the *S*-palmitoylation machinery, in non-target tissues.

### 2.3 Expression of the genes that regulate *S*-palmitoylation in human cancer cell lines

**FIGURE 3:**
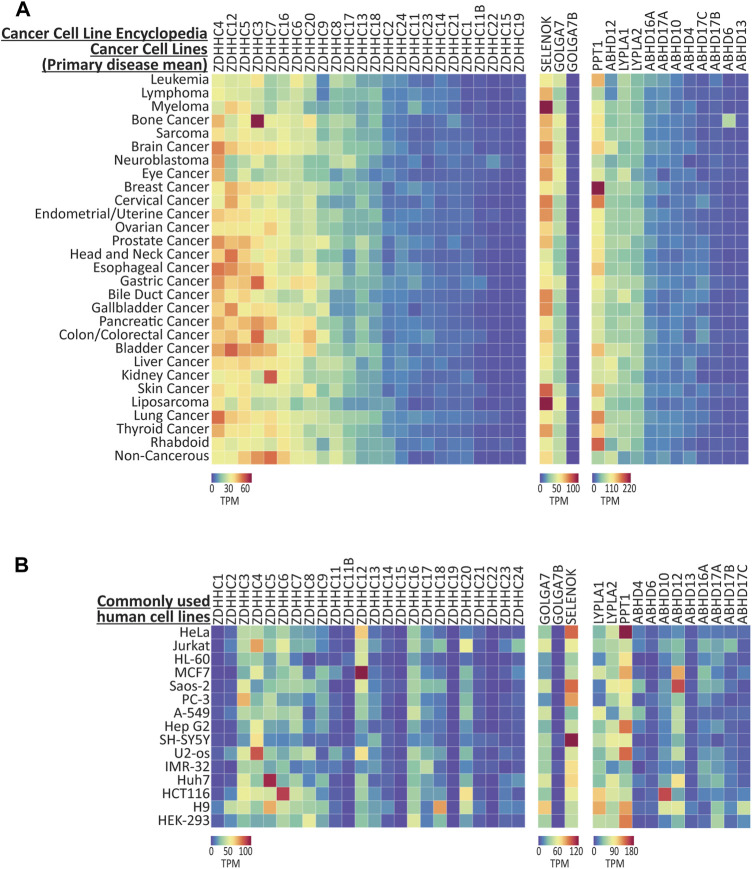
Expression of the genes that regulate palmitoylation from CCLE cancer cell lines. **(A)** Heatmap showing RNAseq expression data for the genes that regulate *S*-palmitoylation, downloaded from CCLE and averaged according to ‘Primary Disease’. Data are included from 1406 cell lines, averaged into 30 primary disease types. **(B)** Expression data for selected human cell lines that are commonly used for basic research. All data were extracted from the CCLE dataset, except the H9 cell line which was found only in the CMP dataset, and HEK-293 that was found in the HPA cell line dataset. Heatmap units: Transcripts per million (TPM). All data available for download on CellPalmSeq.

Cancer cell lines are heavily utilized as preclinical model systems for cancer research (Barretina et al., 2012), and have been critical for the discovery of many fundamental processes in cell biology, such as the characterization of the signaling machinery engaged by T-cell receptors using tumor derived Jurkat cells (Abraham and Weiss, 2004). Importantly, *S*-palmitoylation is known to play a role in the activation of oncogenic signaling networks, and the enzymes that regulate *S*-palmitoylation are known to be dysregulated in a number of cancers (Yeste-Velasco et al., 2015; Anderson and Ragan, 2016; Fhu and Ali, 2021). We next investigated two large scale cancer RNAseq compendia, the Cancer Cell Line Encyclopedia (CCLE; 1406 cell lines; Barretina et al., 2012; Ghandi et al., 2019) and Cell Model Passports (CMP; 442 cell lines; Garcia-Alonso et al., 2017; Picco et al., 2019), that measured gene expression across several hundred cancer cell lines. Expression data (transcripts per million; TPM) were again downloaded for the genes that regulate *S*-palmitoylation and are available for interactive plotting on CellPalmSeq. To provide an overview of differential enrichment of these genes, we averaged expression according to the primary disease tissue (or cancer type) from which the cell lines were isolated, and plotted heatmaps that revealed highly heterogeneous expression of the genes that regulate *S*-palmitoylation across cancer cell lines for both the CCLE (Figure 3A) and CMP datasets (Supplementary Figure S3). The heatmap columns were ranked by descending averages to determine which genes had the broadest expression across cancer cell lines. For the ZDHHC enzymes, *ZDHHC4, ZDHHC5, ZDHHC6, ZDHHC7, ZDHHC12, ZDHHC16*, *and ZDHHC20* were the highest expressing ZDHHC enzymes across cell lines in both datasets, although their ranked expression differed in each set. This is likely due to differences in the number of unique cell lines selected for testing, as only 161 out of 442 cell lines from the CMP dataset were also tested in the CCLE panel. Conversely, expression of *ZDHHC15* and *ZDHHC19* was very low in the majority of cell lines in both studies. Some patterns of expression were consistent across the two datasets (Figure 3A; Supplementary Figure S3A), including high expression of *ZDHHC3* and *ABHD6* in bone cancer derived cell lines, particularly those derived from Ewing’s sarcoma tumors. *ZDHHC7* was elevated in kidney cancer cell lines in both datasets, and *PPT1* was highly expressed in cell lines from breast cancer tumors. Conversely, *ZDHHC9* expression was markedly lowest in cell lines derived from leukemia and lymphoma tissues in both datasets. Because human cancer cell lines often share many transcriptomic similarities with primary tumors (Barretina et al., 2012), this resource to examine the expression patterns of the genes that regulate *S*-palmitoylation across cancer cell lines and cancer types may enable hypothesis generation regarding how *S*-palmitoylation is regulated in certain cancers.

We next plotted the expression data for *S*-palmitoylation associated genes across several of the most commonly used human cell lines (Figure 3B). Although these cell lines have been used extensively for cancer research, many have been utilized outside of this field for fundamental research into cell biology and drug discovery (Supplementary Table S1). Furthermore, the HEK-293 human cells are included as they are commonly used in basic research, although this cell line was derived embryonic kidney cells transformed with adenovirus, and not from tumor tissue (Graham et al., 1977). Heterogeneous expression of the palmitoylating enzymes, their accessory proteins and the depalmitoylating enzymes was again observed across these cell lines, highlighting the importance of considering the expression of this family of enzymes when choosing cell lines as model systems for research on processes that are regulated by *S*-palmitoylation.

### 2.4 Correlation of RNA and protein expression for genes that regulate *S*-palmitoylation in human cancer cell lines

**FIGURE 4:**
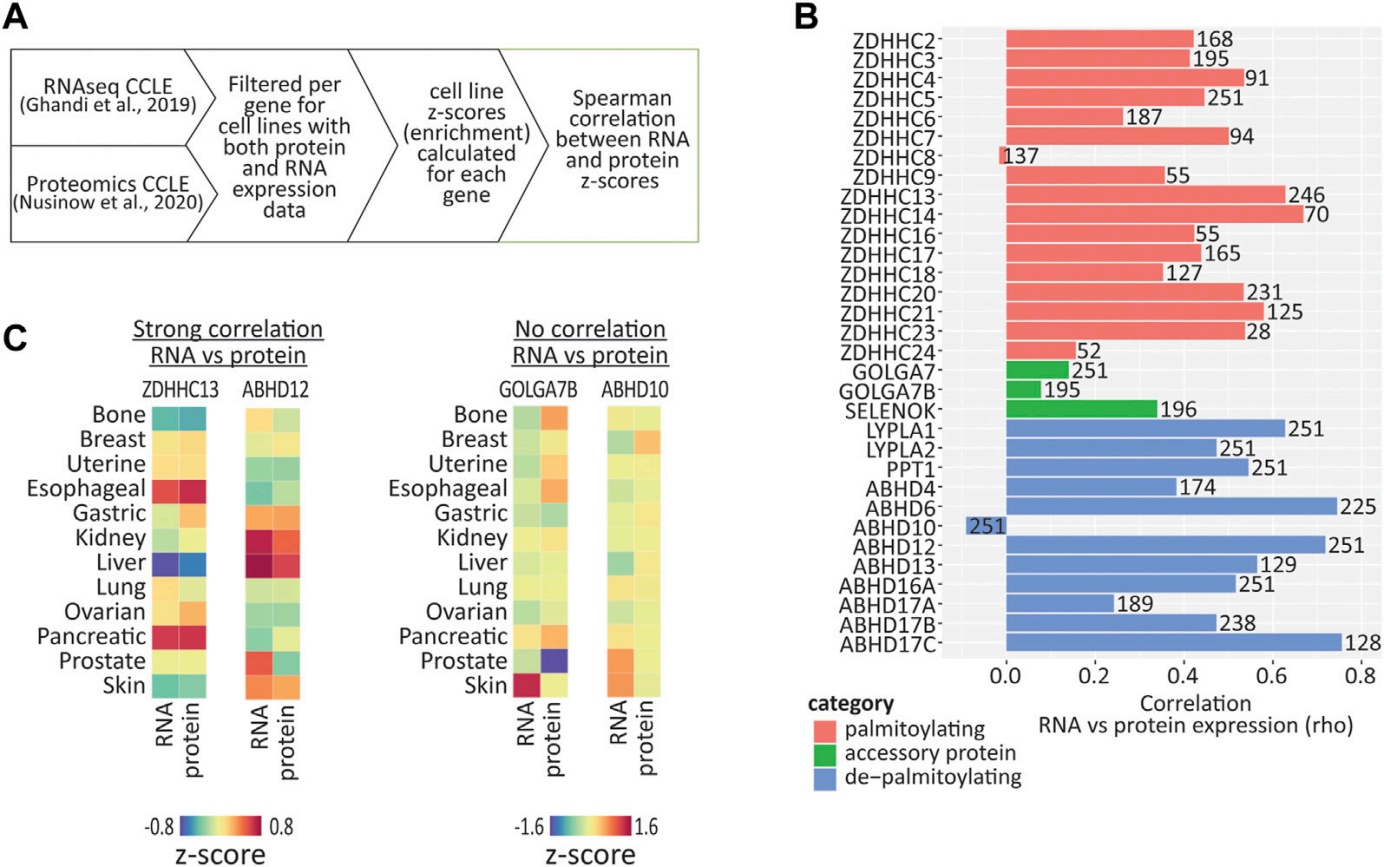
Correlation between RNA and protein expression using cancer cell line data. **(A)** Diagram illustrating how expression values were converted to z-scores and then correlation values calculated, using CCLE RNAseq and proteomic data. **(B)** Graph of Spearman correlation values calculated from expression z-scores across available CCLE cell lines, for RNA vs. protein. Number on each bar represents the number of cell lines (in which both RNA and protein were detected) used for each correlation calculation. **(C)** Heatmap showing enrichments of both RNA and protein across cancer type clusters for selected palmitoylating and depalmitoylating enzymes. Heatmap units: z-score.

Although RNA expression patterns are a useful tool to predict the protein expression in a given cell type, the correlation between RNA and protein expression can be poor, due to post-translational protein processing and degradation (Vogel and Marcotte, 2012). To determine which of the *S*-palmitoylation associated genes have RNA expression patterns that are maintained at the protein level in human cancer cell lines, we compared RNA (Ghandi et al., 2019) and protein (mass spectrometry proteomics; Nusinow et al., 2020) expression data, both generated by the CCLE study (Figure 4A). We found 251 cell lines that were present in both datasets, however, not all of our proteins of interest were detected across all cell lines. We therefore only performed correlations on cell lines that had values for both RNA and protein for a given gene, and converted the expression values into z-scores across these cell lines in order to perform a comparison of cell line enrichments (Supplementary Table S2). In addition, several genes were not detected at all at the protein level, including *ZDHHC1, ZDHHC11, ZDHHC11B, ZDHHC12, ZDHHC19* and *ZDHHC22*. We found that 21 out of the 33 genes examined showed a moderate or strong positive correlation (R > 0.4; *p* < 0.001) between RNA and protein z-scores (Figure 4B), indicating that RNA expression patterns are predictive of protein abundance for the majority of *S*-palmitoylation associated genes. There were however several notable examples of very poor correlation, including *ZDHHC8*, *GOLGA7B* and *ABHD10*.

Closer examination of examples of genes with high correlations between RNA and protein revealed that certain genes were either enriched or depleted within certain cancer cell line clusters (Figure 4C). For example, ZDHHC13 RNA and protein were highly enriched in esophageal and pancreatic cancer cell lines, and depleted in liver cancer cell lines, while ABHD12 was enriched in kidney and liver cancer cell lines. Conversely, GOLGA7B and ABHD10 showed very poor correlation, indicating that caution should be taken if attempting to infer expression patterns of these genes from RNA expression patterns alone.

### 2.5 Expression of the genes that regulate *S*-palmitoylation in commonly used non-human cell lines

Numerous non-human cell lines are widely utilized in basic and pre-clinical research and have been used for the study of basic cell biology, drug toxicity, gene therapy and vaccine production (Verma et al., 2020). However, to our knowledge no single resource exists that has performed a multi-cell line RNAseq study on the most commonly used non-human cell lines. We therefore turned to the Gene Expression Omnibus (GEO; https://www.ncbi.nlm.nih.gov/geo/), a database repository of high throughput gene expression data, to manually curate available RNAseq data for the several of the most commonly used cell lines including: NIH/3T3, PC12, MDCK, BHK21, CHO, VeroE6, and Calu3 (Supplementary Table S3). We curated studies according to the following criteria: i) datasets were associated with a peer reviewed publication; ii) control samples had been tested with minimal manipulation and iii) the source of the cell line used was well defined or the cell line had been authenticated by short tandem repeat (STR) profiling. We then reanalyzed control samples from these curated datasets from the raw reads using the same analysis pipeline. The predicted ensembl (https://ensembl.org) gene set for each species was used to extract the *S*-palmitoylation genes but as expected, not all genes we previously analyzed in human are identified in each species, therefore only those identified are included. All of these datasets are available for interactive visualization and download on CellPalmSeq.

**FIGURE 5:**
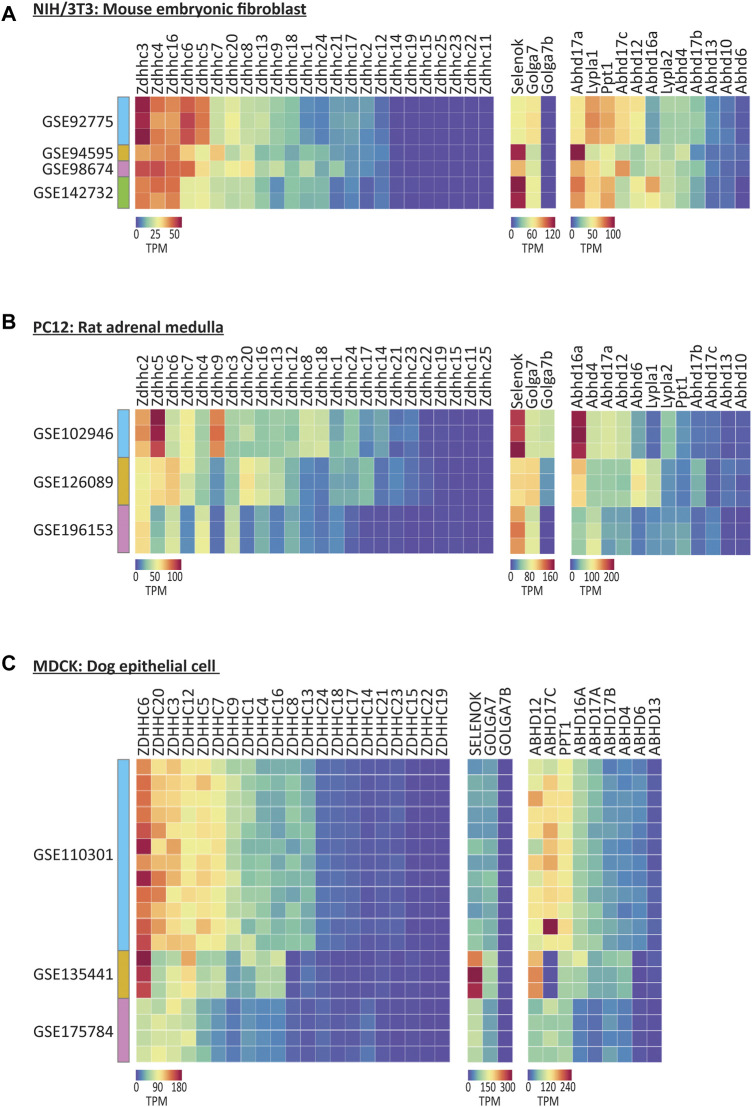
Expression of the genes that regulate S-palmitoylation in selected commonly used non-human cell lines. **(A)** Heatmap of RNAseq expression data for the genes that regulate *S*-palmitoylation in control/untreated samples from mouse NIH/3T3 cell line, downloaded from selected datasets on GEO (labelled with GEO accession numbers). *N* = 1–3 control samples per dataset. Units = Transcripts per million (TPM). **(B)** As A but for Rat PC12 cell line samples. *N* = 3 samples per cell dataset. **(C)** As A but for dog MDCK cell line samples. *N* = 3–12 samples per dataset.

We began with the mouse NIH/3T3 cell line, which is one of the most commonly used embryonic fibroblast lines with 8,826 citations on PubMed.gov. We found that expression patterns for the genes that regulate *S*-palmitoylation were very similar across four independent studies, particularly for the ZDHHC enzymes, despite the cell lines originating from three independent sources (Figure 5A; Supplementary Table S3). *Zdhhc3*, *Zdhhc4* and *Zdhhc16* were the highest expressing ZDHHCs across studies, while numerous ZDHHCs were not detected at all. *Abhd17a*, *Lypla1*, and *Ppt1* were the highest expressing depalmitoylating enzymes, and of the accessory proteins, all were detected except *Golga7b*.

The expression patterns were markedly different in PC12 cells derived from rat adrenal medulla, that share many common features with neuronal cells (Figure 5B; Westerink and Ewing, 2008). Notably, *Zdhhc2* and *Zdhhc5* were on average the highest expressing ZDHHCs across three studies analyzed. Overall, less cross study similarity was observed, highlighting the potential transcriptional heterogeneity in this cell line when derived from different sources. Unlike NIH/3T3 cells, *Golga7b* expression was detected in two of the three studies of PC12 cells, consistent with the neuronal expression for this accessory protein (Wild et al., 2022).

Analysis of MDCK cells derived from canine epithelial cells (Dukes et al., 2011), which have been used in broad applications in biological research and vaccine production, and have been cited over 11,000 times on PubMed, revealed again differences in the highest expressing enzymes, this time with *ZDHHC6* and *ZDHHC20* having the highest expression on average of the ZDHHCs and *ABHD12* being the highest expressing depalmitoylating enzyme (Figure 5C).

Finally, we analyzed several of the most commonly used cell lines for SARS-CoV-2 research, as *S*-palmitoylation of the SARS-CoV-2 spike protein is essential for viral membrane fusion and infectivity (Wu et al., 2021). We collated data for VeroE6 from African Green Monkey and Calu-3, which is a human derived cancer cell line. These cell lines are often selected for SARS-CoV-2 research due to their expression of the ACE-2 receptor, which is required for SARS-CoV-2 entry into the cell (Kumar et al., 2021). We plotted the average of VeroE6 and Calu-3 datasets collated from GEO, along with data from several human cell lines that have been used to identify the palmitoylating enzymes for SARS-CoV-2 spike protein (Supplementary Figure S4; Mesquita et al., 2021; Puthenveetil et al., 2021; Ramadan et al., 2022). We found that expression of the putative ZDHHC enzymes that palmitoylate the SARS-CoV-2 spike protein (*ZDHHC3*, *ZDHHC5*, *ZDHHC8*, *ZDHHC9* and *ZDHHC20*; red bars Supplementary Figure S4) differed across the cell lines, which could alter *S*-palmitoylation of the spike protein, and in turn viral infectivity. Overall, these results reveal how understanding the composition of the *S*-palmitoylation related machinery in a cell line might aid with cell line selection, the design of experiments and the interpretation of results, when using cell lines to study important biological processes that depend on protein *S*-palmitoylation.

## 3 Discussion

### 3.1 Heterogeneous expression of the genes that regulate *S*-palmitoylation across human cell types

Protein *S*-palmitoylation is a dynamic regulator of numerous signaling pathways that are critical for normal cell function (Main and Fuller, 2022). It is estimated that over 10% of proteins in the human proteome are *S*-palmitoylation substrates (Blanc et al., 2015), therefore the majority of cellular signaling pathways are likely to be regulated by this post translational modification. The list of *S*-palmitoylated proteins that are known to be critically involved in human diseases is expanding (Fraser, 2019), and therefore understanding the unique makeup of the *S*-palmitoylation machinery in a given cell type is essential when studying processes that are regulated by *S*-palmitoylation.

Recent advances in RNAseq technologies have enabled the detailed characterization of cellular transcriptomes at a reduced monetary cost, with increased sequencing depth and single cell resolution. This has been accompanied by an increase in the number of studies performed on human tissue, and an appreciation for the importance of open data that can be accessed by the research community in order to reveal novel insights into human physiology and disease. Here, we capitalized on these advances by using open data to investigate the expression patterns of the genes that regulate *S*-palmitoylation in human single cell types, tissues and cancer cell lines, and created an interactive resource to give other researchers easy access to this data.

When examining the distribution of the genes that regulate *S*-palmitoylation in the various cell types from isolated human tissues, we observed that many cell types express a unique compliment of palmitoylating and depalmitoylating enzymes. Because members of this family of proteins are targeted to different subcellular organelles (Globa and Bamji, 2017), and this subcellular targeting can influence the localization and trafficking of substrates (Solis et al., 2022), differential expression of these enzymes and accessory proteins will have a profound influence on substrate function in different cellular contexts. For example, the ZDHHC5 accessory protein GOLGA7B, which is known to regulate ZDHHC5 cell membrane localization, was expressed in neuronal cells, but not detected in cardiomyoctyes. Because ZDHHC5 is known to have an important role in both of these cell types (Woodley and Collins, 2021), this differential expression of GOLGA7B could alter the function of ZDHHC5 in these different cellular contexts.

Drugs that target the ZDHHC palmitoylating enzymes are a potential avenue to treat disorders that are associated with dysregulated *S*-palmitoylation (Fraser, 2019). Efforts are currently underway to develop drugs that selectively target individual ZDHHC enzymes (Salaun et al., 2022). However, a large number of proteins can be *S*-palmitoylated by more than one ZDHHC enzyme, resulting in functional redundancy within this enzyme family that may reduce the therapeutic potential of selective inhibition of individual ZDHHCs. Examination of the relative expression of functionally related ZDHHC enzymes within a given cell type using CellPalmSeq will therefore facilitate predictions of the therapeutic efficacy of selective ZDHHC inhibitors. Furthermore, this data will enable researchers and clinicians to identify ZDHHCs with enriched expression in target cell types and tissues, and better predict off-target effects of selective inhibitors in non-diseased tissues.

### 3.2 Expression patterns of the genes that regulate *S*-palmitoylation in cancer cell lines may give insight into cancer pathologies

The role of *S*-palmitoylation in cancer pathology is now well established (Anderson and Ragan, 2016; Lin et al., 2017; Ko and Dixon, 2018; Lobo, 2019). A recent study identified 299 cancer driving genes (Bailey et al., 2018), of which 78 are substrates for *S*-palmitoylation (Ko and Dixon, 2018). Furthermore, altered expression of almost all of the 24 ZDHHC enzymes is associated with positive or negative prognosis in various cancers (Ko and Dixon, 2018). Interestingly, several of the expression patterns of the genes that regulate *S*-palmitoylation that we found here in certain cancer cell lines have also been previously reported in the literature. For example, we observed elevated expression of *ABHD6* in cell lines with a bone cancer origin, particularly those from Ewing’s sarcoma cell lines. High expression of *ABHD6* in Ewing’s sarcoma cell lines were found previously (Max et al., 2009), while another study found a carcinogenic role for ABHD6 in metastatic seeding of murine pancreatic ductal adenocarcinoma cells *in vivo* (Grüner et al., 2016). Although ABHD6 has an important function in degrading the endocannabinoid 2-arachidonoylglycerol, the role of ABHD6 as a depalmitoylating enzyme has not yet been studied, despite being potently inhibited by depalmitoylating enzyme inhibitors HDFP and PalmB (Lin et al., 2017). We also observed across studies highly elevated expression of *ZDHHC7* in kidney cancer cell lines and *PPT1* in breast cancer cell lines, opening potential avenues for research into the role of these enzymes in these cancers. Extensive RNAseq datasets are now available that have characterized gene expression in patient isolated tumors. Investigation of the expression patterns of the genes that regulate *S*-palmitoylation from these data will further expand any insight into the role of this family of proteins within certain cancer types.

While RNAseq expression patterns are useful for the prediction of protein abundance in certain cell types, RNA and protein expression do not often correlate well due to post translational protein processing (Vogel and Marcotte, 2012). Furthermore, the sensitivity of proteomic assays currently lags behind that of RNA sequencing, as unlike RNA, protein cannot be amplified to enhance detection. Here we took advantage of the availability of both RNAseq and proteomic datasets covering a large number of cancer cell lines, which allowed us to perform an in-depth study of the correlation between RNA and protein expression for the majority of the genes that regulate *S*-palmitoylation. Although we observed moderate or strong positive correlation for the majority of genes tested, there are notable exceptions including ZDHHC8, ZDHHC24, GOLGA7, GOLGA7B, and ABHD10 which correlated poorly. Particular caution is therefore advised when inferring protein abundance from RNA expression for these proteins, which may be subject to more extensive post transcriptional regulation.

### 3.3 Consideration of the expression of the genes that regulate *S*-palmitoylation when using cellular model systems to study biology and disease

We have used examples of non-human cell lines and the cell lines that are most commonly used for SARS-CoV-2 research to demonstrate that heterogeneity in ZDHHC expression should be taken into account when choosing cell lines for research and interpreting results, particularly when studying *S*-palmitoylation dependent processes. For example, when selecting cell lines for SARS-CoV-2 research, we show that the expression of the putative ZDHHC palmitoylating enzymes for the spike protein differed across cell lines that are commonly used for this research. In addition, consideration of the similarities in expression profiles between laboratory cell lines and the endogenous cell types being studied will also be beneficial. For studies on SARS-CoV-2 infection, which predominantly affects respiratory ciliated cells (Hou et al., 2020; Robinot et al., 2021), the single-cell expression profile for this cell type detailed in Figure 1, combined with the expression profiles of the popular cell lines used for SARS-CoV-2 research in Supplementary Figure S4, can guide the selection of the most suitable cell line.

Finally, we found that certain cell lines were more consistent in their expression of the ZDHHC enzymes, when derived from multiple sources, and researchers are recommended to sequence their own cell lines for most accurate assessment of gene expression. When available, we believe that the large panel screens of multiple cell lines that use systematic and standardized culture and RNAseq protocols (such as CCLE and CMP) are the most reliable resource for assessing relative trends in expression profiles of these genes.

CellPalmSeq will be an invaluable resource that will enable researchers and clinicians to easily interrogate the expression patterns of the *S*-palmitoylation machinery in the human body and cell line model systems, and therefore will facilitate research into the role of *S*-palmitoylation in cellular biology and disease.

## 4 Materials and methods

### 4.1 Data processing for CellPalmSeq

For Human Protein Atlas, normalized single-cell RNAseq data collated from 26 datasets (www.proteinatlas.org/download/rna_single_cell_type.tsv.zip) were directly downloaded from (www.proteinatlas.org/about/download) and data were extracted (nTPM) for the genes that regulate *S*-palmitoylation.

For Cancer Cell Line Encylclopedia (CCLE), RNAseq data were downloaded from https://depmap.org/portal/download/ (file CCLE_expression.csv version 22Q2) and averaged by cell lineage and primary disease. Proteomic data were downloaded from https://gygi.hms.harvard.edu/publications/ccle.html (file Supplementary Table S2_Protein_Quant_Normalized.xlsx). Correlations between RNAseq and proteomic data were performed with R using the Spearman method.

For Cell Model Passports (CMP), data were downloaded from https://cellmodelpassports.sanger.ac.uk/downloads (file rnaseq_all_20220624.zip) and averaged by tissue and cancer type.

For datasets downloaded from the GEO, the reference transcriptome of each species for which cell lines were analyzed from raw sequencing reads was acquired from https://ensembl.org. The RNAseq reads were downloaded from the Gene Expression Omnibus (https://www.ncbi.nlm.nih.gov/geo/), the series accession numbers and individual sample numbers are listed in Supplementary Table S3. For each sample, RNAseq expression values (TPM) at the gene level were obtained using kallisto (Bray et al., 2016) with the appropriate reference transcriptome by summing the TPM values of individual isoforms.

### 4.2 Heatmap creation for CellPalmSeq

All plots for the CellPalmSeq database were generated using curated RNA sequencing datasets. Python 3 and Javascript scripts were used with the plotting library Bokeh to generate the interactive heatmaps to display and compare these datasets on the CellPalmSeq website (Bokeh Development Team, 2018).

### 4.3 Data presentation

Heatmaps within the manuscript were plotted in Displayr (https://www.displayr.com). Bar charts were plotted in GraphPad Prism 9.2.0 (San Diego, CA, and United States).

## Data Availability

Publicly available datasets were analyzed in this study. This data can be found here: https://cellpalmseq.med.ubc.ca.
