# Supplementary figures and images for: CellPalmSeq: A curated RNAseq database of palmitoylating and de-palmitoylating enzyme expression in human cell types and laboratory cell lines
Source: Front Physiol. 2023 Jan 24;14:1110550. doi: 10.3389/fphys.2023.1110550 (PMC9904442; doi:10.3389/fphys.2023.1110550)

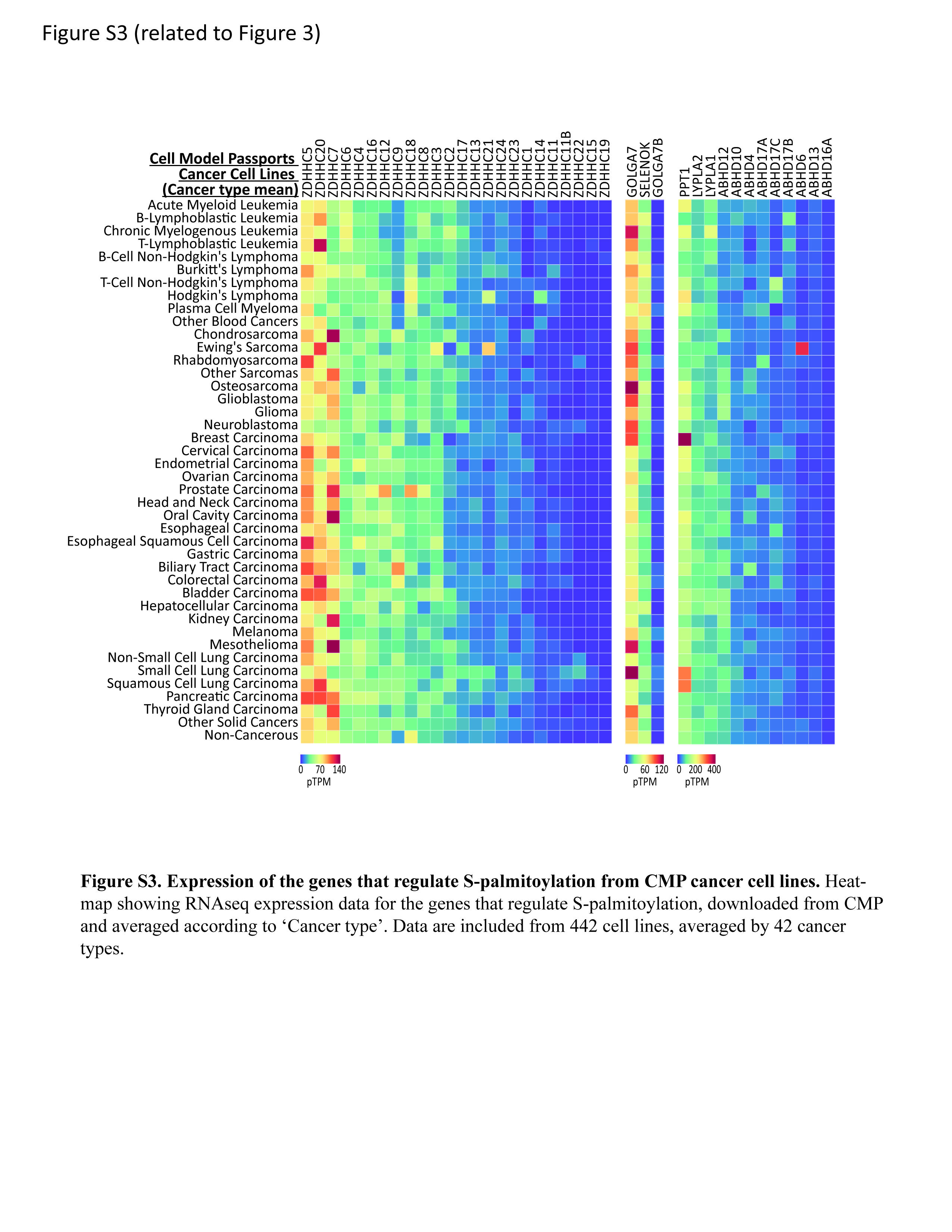

Supplement: Supplementary file 3 [file Image3.tif]

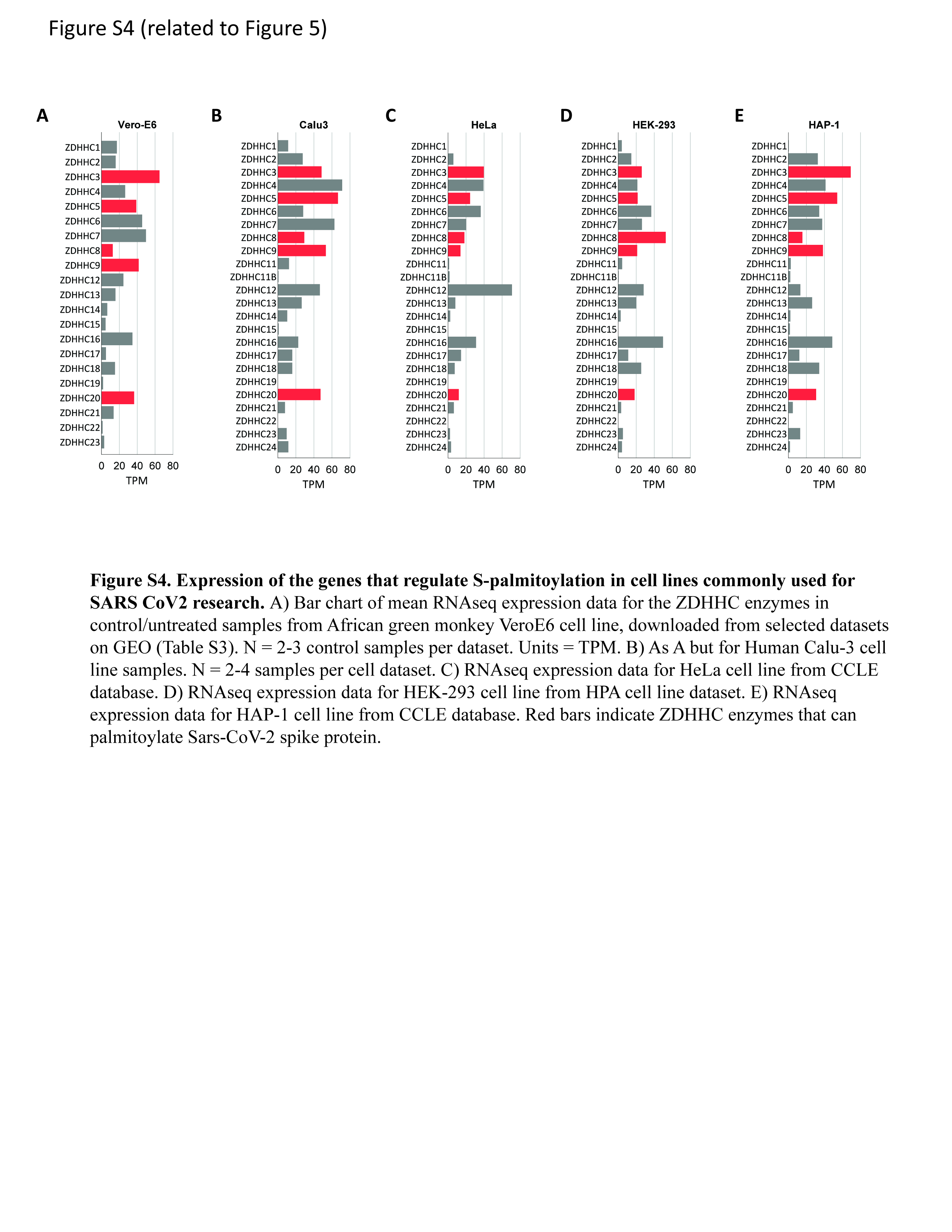

Supplement: Supplementary file 4 [file Image4.TIF]

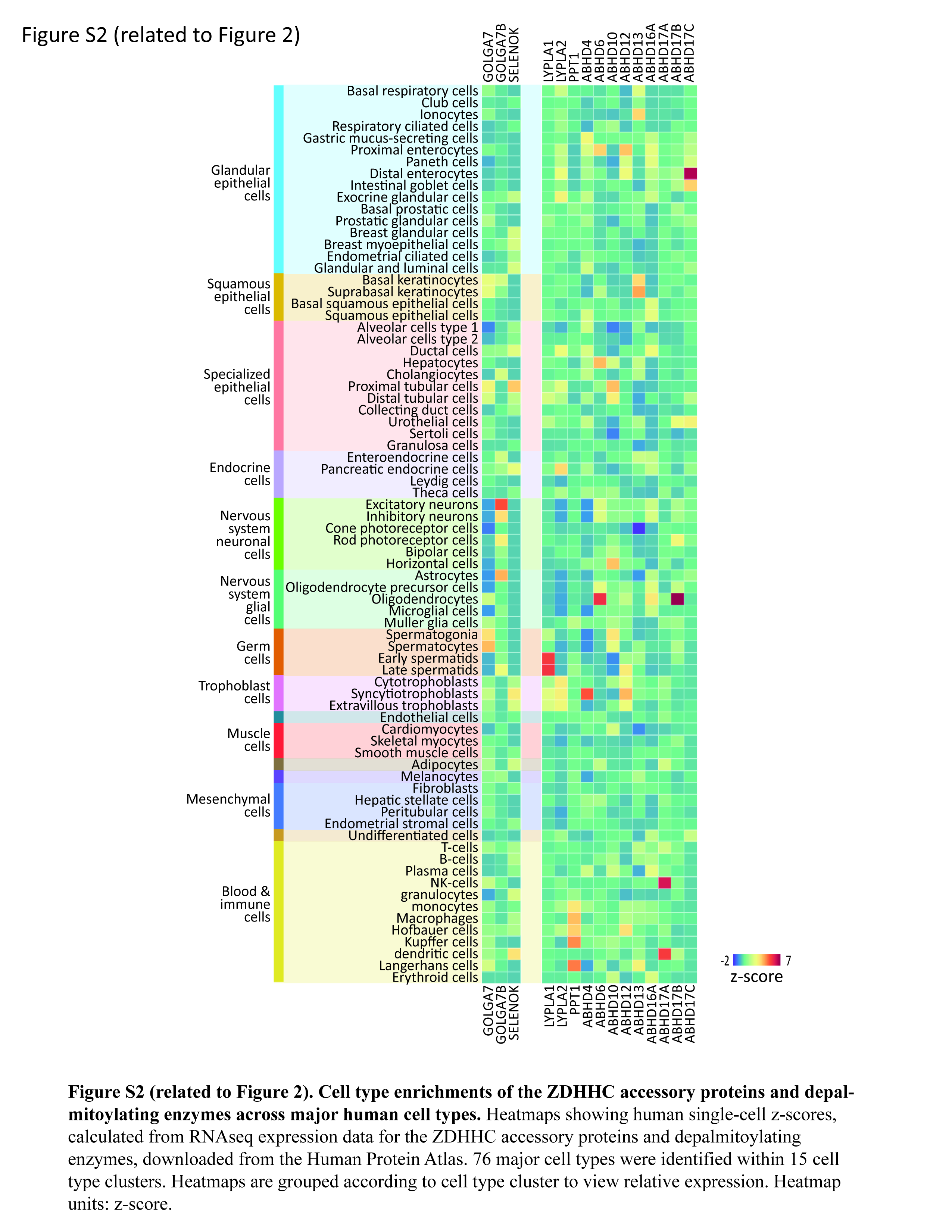

Supplement: Supplementary file 5 [file Image2.TIF]

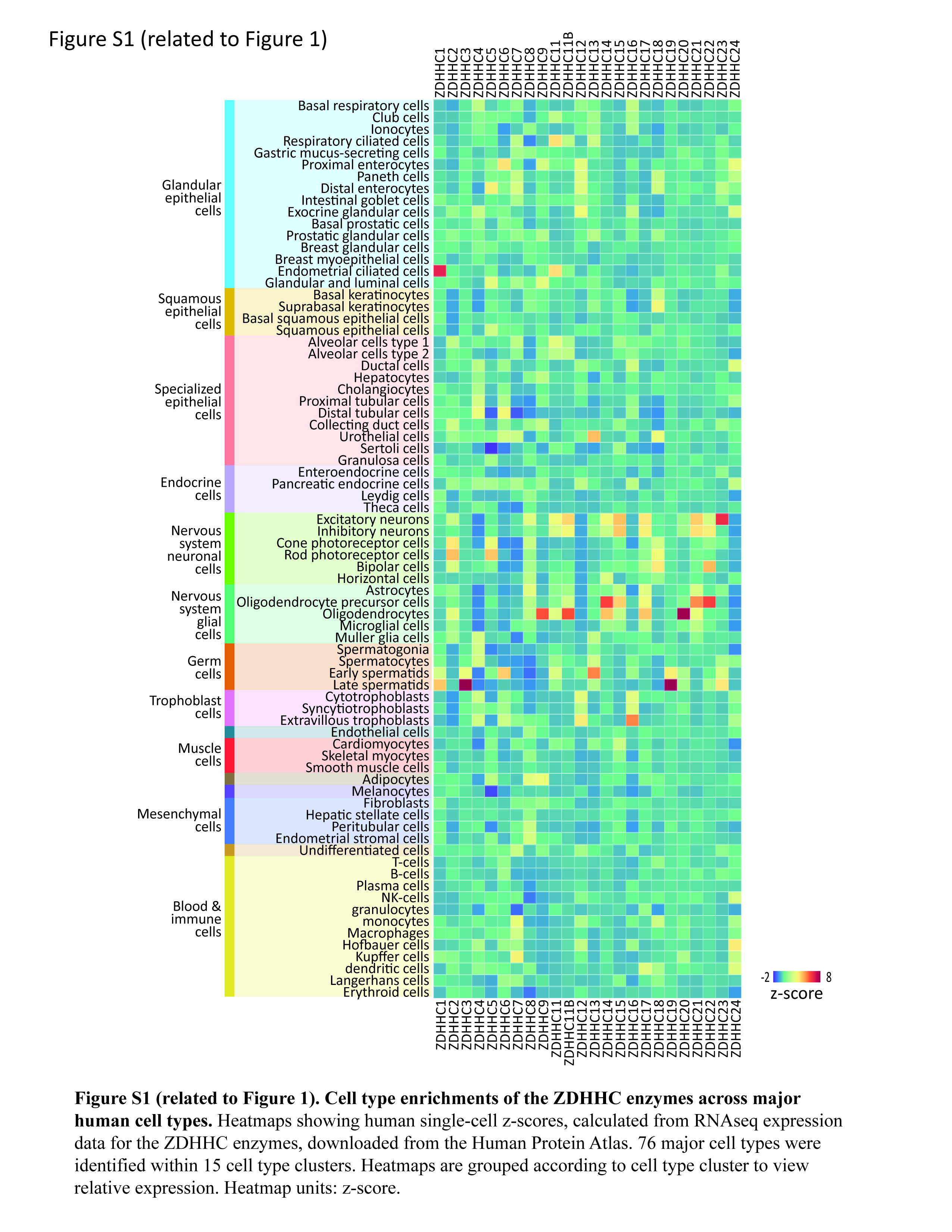

Supplement: Supplementary file 6 [file Image1.TIF]
